# Progression and influencing factors of knee osteoarthritis based on a multi-state Markov model: Data from OAI

**DOI:** 10.1016/j.ocarto.2025.100703

**Published:** 2025-11-13

**Authors:** Aerman Nuer, Yasi Yang, Boran Sun, Yunhan Wang, Wenbo Xiao, Lei Wang, Shu Wang, Wenli Lu

**Affiliations:** a Department of Epidemiology and Statistics, School of Public Health, Tianjin Medical University, Tianjin, China; b School of Biomedical Engineering and Technology, Tianjin Medical University, Tianjin, China; c Institute of Biomedical Engineering, Chinese Academy of Medical Science & Peking Union Medical College, Tianjin, China; d Tianjin Academy of Traditional Chinese Medicine Affiliated Hospital, Tianjin, China; e National Clinical Research Center for Chinese Medicine Acupuncture and Moxibustion, Tianjin, China; f Tianjin Institution of Acupuncture and Moxibustion, Tianjin, China

**Keywords:** Knee osteoarthritis, Multi-state Markov model, States transition, Risk factors

## Abstract

**Background:**

Knee osteoarthritis (KOA) is a chronic disorder marked by progressive cartilage loss and functional decline. Current classifications miss bidirectional transitions, particularly in early and late stages, hindering early intervention.

**Methods:**

Participants aged 45–79 years from the Osteoarthritis Initiative (OAI) were analyzed over eight years, classifying KOA states as normal, early-KOA, radiographic KOA (rKOA), and end-stage KOA (es-KOA) using Kellgren-Lawrence (K-L) grades, symptoms, and patient-reported outcomes. A Multi-state Markov (MSM) model evaluated state transitions and risk factors.

**Results:**

The study comprised 2043 individuals (55.0 ​% female, 85.9 ​% White) with a total of 13,997 records of KOA state assessment. Of the individuals currently classified as early-KOA, 34.0 ​% returned to normal state, 60.9 ​% remained in early-KOA, and 5.1 ​% progressed to rKOA or es-KOA at next follow-up. The transition intensity from early-KOA to rKOA (0.05, 95 ​% CI: 0.04–0.06) was 2.6 times greater than that from normal to rKOA (0.02, 95 ​% CI: 0.01–0.02). The longest sojourn time was observed in rKOA, with a mean of 15.17 years. In covariate analysis, progression risk increased with obesity (HR: 2.57, Normal to rKOA), poor contralateral knee condition (HR: 3.68, Normal to rKOA), and depressive symptoms (HR: 2.16, rKOA to es-KOA). Better physical function reduced risk (HR: 0.65, Normal to early-KOA).

**Conclusion:**

This study reveals dynamic KOA transitions, with early-KOA and es-KOA showing recovery potential. Identifying risk factors like obesity and contralateral knee condition offers opportunities for targeted interventions to slow progression and improve joint health, emphasizing early management's role in KOA care.

## 1 Introduction

Osteoarthritis (OA) is a common chronic joint disorder marked by cartilage degeneration and changes in subchondral bone and synovium, impairing quality of life [1]. Its global incidence is rising [2], with the knee being the most commonly affected site [3], and the resulting joint damage is irreversible [4]. According to the Global Burden of Disease 2021 report, approximately 595 million individuals worldwide were affected by OA in 2020—a 132.2 ​% increase since 1990—including 368 million with knee OA, predominantly among older adults. OA contributes to 2.3 ​% of global years lived with disability [5,6].

The late-stage diagnosis of KOA limits the opportunity to modify the natural course of the disease during the preclinical phase, thereby hindering the restoration of joint homeostasis and the prevention of joint dysfunction [7]. Current classification systems focus on structural changes but fail to capture the heterogeneity of symptom trajectories and the reversibility in the early stages. This gap underscores the need for a defined preclinical state, prior to irreversible degenerative changes, where interventions could halt disease progression. Research on KOA transitions, particularly the shift from preclinical to symptomatic phases [8], highlights the importance of identifying this window. Therefore, there is a need for a state in which degenerative changes have not yet occurred and reversibility is still possible, allowing for early intervention [9,10].

Multi-state Markov (MSM) models, widely applied in chronic disease research[[11], [12], [13]], offer a robust framework to analyze such dynamic transitions. These models quantify transition intensities, sojourn times, and covariate effects, providing insights into disease progression and recovery patterns. Currently, although the MSM model is applied to KOA [8], a comprehensive understanding of the transitions linked to its early and end-stages remains elusive. Additionally, comorbidities such as obesity and contralateral joint pathology increase the risk of KOA. Obesity is a key risk factor for the onset, progression, and prognosis of KOA, with evidence demonstrating a dose-dependent relationship. A meta-analysis indicated that each 5 ​kg/m^2^ increase in body mass index (BMI) is associated with a 35 ​% higher risk of KOA [14]. This study employs an MSM model to describe the dynamic transitions between different stages of KOA, with the aim of uncovering the progression and transitions of KOA, as well as identifying modifiable risk factors that influence these transitions. By integrating longitudinal radiographic and clinical data, it provides a nuanced understanding of KOA progression, emphasizing the opportunities for early intervention and personalized management strategies.

## 2 Methods

### 2.1 Study population

The data for this study were derived from the Osteoarthritis Initiative (OAI), a publicly available, longitudinal, multi-center cohort study funded by the National Institutes of Health. The OAI includes detailed information on 4796 participants aged 45–79 years and was established to facilitate a better understanding of the prevention and treatment of knee OA (KOA). The study protocol, amendments, and informed consent documentation were reviewed and approved by the local institutional review boards of all participating centers. Detailed information about the OAI and access to its data are available at https://nda.nih.gov/oai.

Of the data available in the OAI, records of Kellgren-Lawrence (K-L) grade are included for seven follow-up visits over the first eight years, so this study incorporates data from these seven follow-up points through the eighth year. The exclusion criteria were as follows: (1) the absence of K-L grade records, (2) death or knee replacement surgery during the follow-up period, and (3) fewer than six follow-up records. The detailed screening process was illustrated in Fig. S1. The comparison of characteristics between enrolled and excluded participants is described in Table S1.

### 2.2 Measurements

In this study, the description of KOA-related definitions was based on radiographic findings according to the K-L grade classification scale. Bilateral, weight-bearing, fixed-flexion, posteroanterior knee radiographs were acquired at baseline and during each follow-up visit in the OAI. Independent central readers, blinded to the sequence of the images, assessed and assigned K-L grades ranging from 0 to 4. The K-L grade was independently assessed by two central experts who were blinded to each other's assessments and to all other study data. Consequently, instances of K-L grade regression were observed. Since joint damage in KOA is considered irreversible, a conservative approach was adopted to address such cases. Specifically, when regression was identified, the K-L grade from the preceding follow-up visit was carried forward. For instance, if the K-L grades for three consecutive visits were recorded as 1, 2, and 1, the grade for the third visit was adjusted to 2.

According to the criteria established by the American Colleg e of Rheumatology [15], knees with a K-L grade below 2 were classified as normal, whereas those with a grade of 2 or higher were defined as having radiographic KOA (rKOA). Building on the K-L grade classification of normal and rKOA, we introduced two additional states: early-KOA and end-stage KOA (es-KOA).

Early-KOA is defined based on a combination of symptomatic, clinical, and radiographic criteria as outlined by Luyten et al. [16]. This definition includes three key aspects: (1) patient-reported outcomes, where at least two of the four Knee Injury and Osteoarthritis Outcome Score subscales score ≤85 ​%, (2) clinical signs, such as joint line tenderness or crepitus, and (3) radiographic findings, characterized by a K-L grade of 0 or 1 on standing, weight-bearing X-rays. The adoption of this validated definition aims to advance understanding of individuals who have not yet developed structural damage and still retain potential reversibility, consistent with the recent perspective advocating a “window of opportunity.” [9].

We adopted the definition of es-KOA proposed and updated by Driban et al. [17], which integrates both symptomatic and radiographic criteria. A knee was considered to have es-KOA if any of the following two conditions were met: severe rKOA (K-L grade ​= ​4) with at least moderate symptoms (Western Ontario and McMaster Universities Arthritis Index [WOMAC] pain ​+ ​disability score > 12) OR (2) K-L grade ​= ​2 or 3 with intense or severe symptoms (WOMAC pain ​+ ​disability score > 22) and persistent knee pain (≥ 3 months in the past year). It integrates radiographic damage with clinically meaningful symptoms, thereby capturing both structural and symptomatic disease burden. This provides a more reliable prediction of future knee arthroplasty [18,19] and also allows knees meeting the es-KOA criteria to potentially regress to a milder state following symptom relief.

To ensure mutually exclusive categorization of states, classification was determined hierarchically. Knees with K-L grade ≥ 2 were first categorized as rKOA, and es-KOA criteria were then applied if symptomatic thresholds were met, early-KOA was assigned only when K–L grade < 2 and the relevant symptomatic criteria were fulfilled.

### 2.3 MSM model

To simulate the dynamic transitions between KOA states, an MSM model was built. The analysis was conducted at the knee joint level, with the model designating each participant's right knee as the target joint for state transitions. There are four states of interest: normal, early-KOA, rKOA, and es-KOA. All four states were modeled as transient, with no absorbing state specified.

**Fig. 1:**
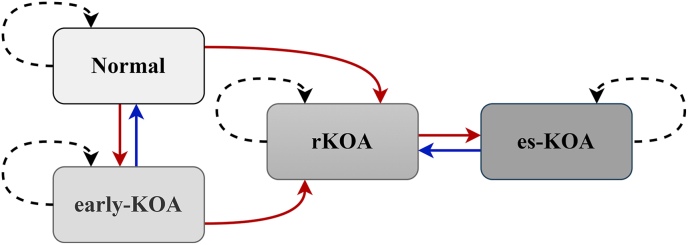
The hypothesis of transitions between different states. Solid red arrows indicate transitions to more advanced states, while dashed black arrows represent the probabilities of remaining in the same state. Blue arrows depict transitions back to less severe states.

The transitions between states are nonlinear, non-sequential, and reversible. Bidirectional movement between normal and early-KOA was allowed to accommodate symptom variability and minor structural changes that may improve or worsen between visits. The early-KOA may progress to rKOA, and due to symptom heterogeneity, the normal state may also develop radiographic changes and progress to rKOA without passing through the early-KOA. It is assumed that neither the normal state nor the early-KOA state can directly progress to the es-KOA, as the progression of KOA is gradual and slow. Furthermore, because the radiographic structural changes of KOA are irreversible, neither rKOA nor es-KOA can revert to the normal or early-KOA states. Bidirectional transition between rKOA and es-KOA was assumed to reflect worsening and partial improvement at advanced stages. Fig. 1 shows possible transitions among these four states.

The Markov framework assumes that the probability of transitioning to a subsequent state depends solely on the current state. Transition intensity denotes the instantaneous per-year rate of moving from one state to another, conditional on the current state. The sojourn time is the expected time spent in a state before leaving it, and transition probabilities summarize, over a prespecified interval, the chance of occupying each state given the current state. Parameter estimation was conducted using maximum likelihood methods, enabling robust and consistent estimation of transition intensities.

### 2.4 Covariates

Covariates included demographic characteristics (age, sex, race, marital status, education, and employment status), lifestyle factors, clinical measurements, and comorbid conditions. Age was categorized into three groups: < 55 years, 55–64 years, and ≥ 65 years. BMI was classified into three categories: normal/underweight (BMI < 25 ​kg/m^2^), overweight (BMI 25–29.9 ​kg/m^2^), and obese/morbidly obese (BMI ≥ 30 ​kg/m^2^). Physical function was assessed using the Physical Component Summary of the SF-12 questionnaire [20], with scores dichotomized at 50 (the population norm). Scores <50 indicated below-average physical health status. Depressive symptoms were evaluated using the Center for Epidemiologic Studies Depression Scale [21], where a score < 16 indicated the absence of depressive symptoms, while a score ≥ 16 suggested their presence. Lifestyle factors included smoking status (never vs. current/former) and alcohol consumption (never, < 7 drinks/week, and ≥ 7 drinks/week). Knee-related conditions encompassed left knee status (normal, early-KOA, rKOA, and es-KOA), family history of knee replacement, and history of right knee injury. Comorbid conditions included diabetes, hypertension, and overall comorbidity burden, assessed using the Charlson Comorbidity Index [22], categorized as 0, 1–2, and ≥ 3. For covariates other than left knee condition, missing values at a given visit were imputed using the most recently observed value carried forward. For participants with no observed values for a given covariate across all visits, the covariate was treated as missing, and the corresponding participant-visit records were excluded.

### 2.5 Statistical analysis

All statistical analyses were performed using R (version 4.3.3), with the MSM model built using the *msm* package (version 1.81). Descriptive statistics were used to summarize baseline characteristics, categorical variables were presented as counts and percentages. The MSM model was implemented in two stages. First, a basic Markov model without covariates was performed to estimate the transition probabilities between predefined states and the mean sojourn time within each state. In the second stage, a univariate model was initially constructed to assess the effects of individual covariates on state transitions. Subsequently, a multivariable model was developed, incorporating covariates identified as having a significant impact on the bidirectional transitions between early KOA and normal states in the univariable analysis, along with demographic factors such as age, sex, and race. This multi-factor model further allowed estimation of subgroup-specific transition probabilities.

We adopted a conservative strategy to replace the missing K-L grades by carrying forward the K-L grade from the previous visit. To evaluate the robustness of the model, we additionally constructed sensitivity analysis models in which es-KOA was defined as the absorbing state, the K-L grades were used without carry-forward, and covariates were included without carry-forward imputation. A two-tailed *P* ​< ​0.05 was considered statistically significant.

## 3 Result

### 3.1 Baseline characteristics of enrolled individuals

A total of 2043 individuals were included in the analysis, and the baseline demographic and clinical characteristics are summarized in Table 1. Of the participants, 55.0 ​% were female and 45.0 ​% male. The mean age was evenly distributed across categories, with 35.2 ​% under 55 years, 33.6 ​% aged 55–64 years, and 31.2 ​% aged 65 years or older. The majority of participants were White (85.9 ​%), while 14.1 ​% identified as other.

**Table 1:** Baseline demographic and clinical characteristics of enrolled participants.

Characteristics (n, %)		Enrolled participants (N ​= ​2043)
Sex	Female	1123 (55.0)
	Male	920 (45.0)
Age	<55	719 (35.2)
	55–64	686 (33.6)
	≥65	638 (31.2)
Race	White	1754 (85.9)
	Other^a^	289 (14.1)
Marital status	Married	1434 (70.2)
	Never married/divorced/separated/widowed	609 (29.8)
Body mass index	Normal/underweight	590 (28.9)
	Overweight	828 (40.5)
	Obese/morbidly obese	625 (30.6)
Education	Low education	226 (11.1)
	Medium education	904 (44.4)
	High education	906 (44.5)
Pay for work	No	657 (32.2)
	Yes	1386 (67.8)
Physical component summary	<50	691 (33.8)
	≥50	1352 (66.2)
Smoking	Never	1592 (78.0)
	Current/former/current but not regular	450 (22.0)
Drinking	Never	784 (38.4)
	<7/week	347 (17.0)
	≥7/week	911 (44.6)
Left knee condition	Normal	1197 (58.6)
	Early-KOA	436 (21.3)
	rKOA	346 (16.9)
	Es-KOA	64 (3.1)
Family knee replacement history	No	1746 (85.5)
	Yes	295 (14.5)
Right knee injury history	No	1492 (73.0)
	Yes	551 (27.0)
Diabetes	No	1918 (93.9)
	Yes	125 (6.1)
Hypertension	No	1084 (53.1)
	Yes	959 (46.9)
Depressive symptoms	No	1875 (91.8)
	Yes	168 (8.2)
Charlson comorbidity index	0	1626 (79.6)
	1–2	374 (18.3)
	≥3	43 (2.1)

Table 1 describes baseline characteristics using each participant's first available observation for each covariate.

a The “other” race category encompasses participants identifying as American Indian/Alaska Native; Asian; Hawaiian or Pacific Islander; Black or African American; More than one race; as well as those whose race was Unknown or not reported, or reported as Other/Other Non-White. This aggregation was performed due to small sample sizes within these individual groups.

### 3.2 Transitions between different states

A total of 304 individuals completed 6 state follow-ups, and 1739 individuals completed 7 state follow-ups, resulting in 13,997 state assessment records. At baseline, 1151 individuals (56.3 ​%) were classified as normal, 439 (21.5 ​%) as early-KOA, 401 (19.6 ​%) as rKOA, and 52 (2.5 ​%) as es-KOA. By the final follow-up, 970 individuals (48.8 ​%) were classified as normal, 323 (16.3 ​%) as early-KOA, 592 (29.8 ​%) as rKOA, and 102 (5.1 ​%) as es-KOA. The distribution of different states during each period are summarized in Table S2.

**Fig. 2:**
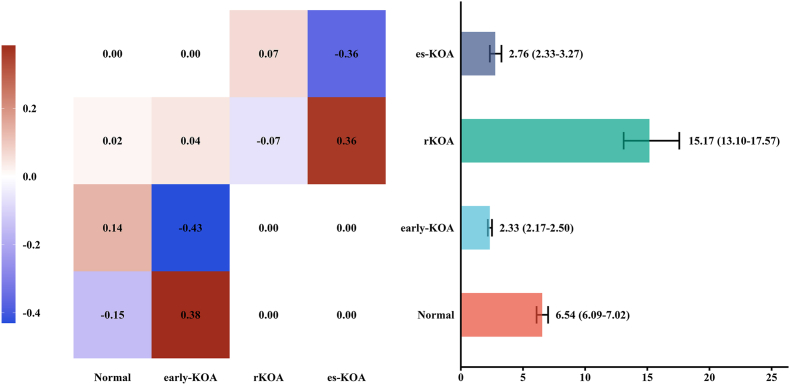
Transition intensity between different states and sojourn time. The transition intensities between states are depicted in the left panel of the figure. Each cell represents the transition intensity from one state to another, with darker colors indicating higher intensity. The right panel represents the sojourn time participants spent in a specific state before transitioning to another.

The transition intensities between different states and the sojourn times were shown in Fig. 2 and Table S4. For individuals in the early-KOA state, the transition intensity to the rKOA state (0.05, 95 ​% confidence interval [CI]: 0.04–0.06), was 2.6 times higher than the transition intensity from the normal state to the rKOA state, which was 0.02 (95 ​% CI: 0.01–0.02). Additionally, the transition intensity from the early-KOA state back to the normal state, 0.38 (95 ​% CI: 0.36–0.42), was 2.8 times higher than the transition intensity from the normal state to the early-KOA state, which was 0.14 (95 ​% CI: 0.13–0.15). The transition intensities and probabilities, with es-KOA as the absorbing state and using the raw K-L grades, are presented in Tables S7–S10.

The observed one-interval transitions are presented in Table S3. Among knees classified as normal at the preceding visit, 11.9 ​% transitioned to early-KOA, 2.3 ​% to rKOA, and 0.2 ​% to es-KOA at the subsequent visit. Among those in the early-KOA state, 34.0 ​% regressed to normal and 3.9 ​% progressed to rKOA.

Individuals had the longest sojourn time in the rKOA state, averaging 15.17 years (95 ​% CI: 13.10–17.57), followed by the normal state at 6.54 years (95 ​% CI: 6.09–7.02), the early-KOA state at 2.33 years (95 ​% CI: 2.17–2.51), and the es-KOA state at 2.76 years (95 ​% CI: 2.33–3.27). Fig. S2 shows the observed and predicted prevalence of each state against time, the model fitted well.

### 3.3 Transition probability of different states

**Fig. 3:**
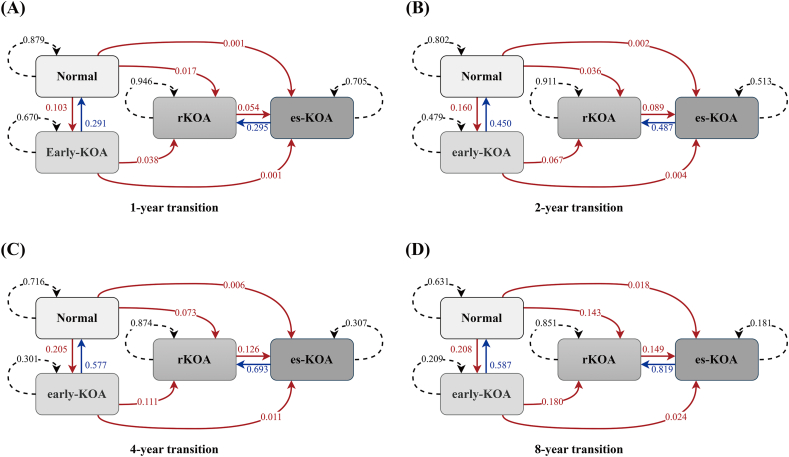
State transition diagrams. State transition diagrams showing probabilities of transitions over different time intervals: (A) 1-year transition, (B) 2-year transition, (C) 4-year transition, and (D) 8-year transition. The numbers along the arrows represent the probabilities of transitioning from one state to another within the specified interval. Solid red arrows indicate transitions to more advanced states, while dashed black arrows represent the probabilities of remaining in the same state. Blue arrows depict transitions back to less severe states.

Fig. 3 estimates the probabilities of state transitions for individuals in different states over observation intervals of 1, 2, 4, and 8 years. Table S4 and Fig. S3 summarize the state transition probabilities across intervals ranging from 1 to 8 years. For individuals in the normal state, the probabilities of remaining in the normal state or transitioning to early-KOA, rKOA, and es-KOA were 0.879, 0.103, 0.017, and 0.001, respectively, over a 1-year observation, and evolved into 0.631, 0.208, 0.143, and 0.018, respectively, over an 8-year observation. For individuals in the early-KOA state, the probabilities of remaining in the early-KOA state or transitioning to normal, rKOA, and es-KOA were 0.670, 0.291, 0.038, and 0.001, respectively, over a 1-year observation, and evolved into 0.209, 0.587, 0.180, and 0.024, respectively, over an 8-year observation. For individuals in the rKOA state, the probabilities of remaining in the rKOA state or transitioning to es-KOA were 0.946 and 0.054, respectively, over a 1-year observation, and evolved into 0.851 and 0.149, respectively, over an 8-year observation. For individuals in the es-KOA state, the estimated probability of remaining in the es-KOA state was 0.705, while the probability of transitioning to rKOA was 0.295 over a 1-year observation, and these probabilities evolved into 0.819 and 0.181, respectively, over an 8-year observation.

### 3.4 Covariate effects on different transitions

**Fig. 4:**
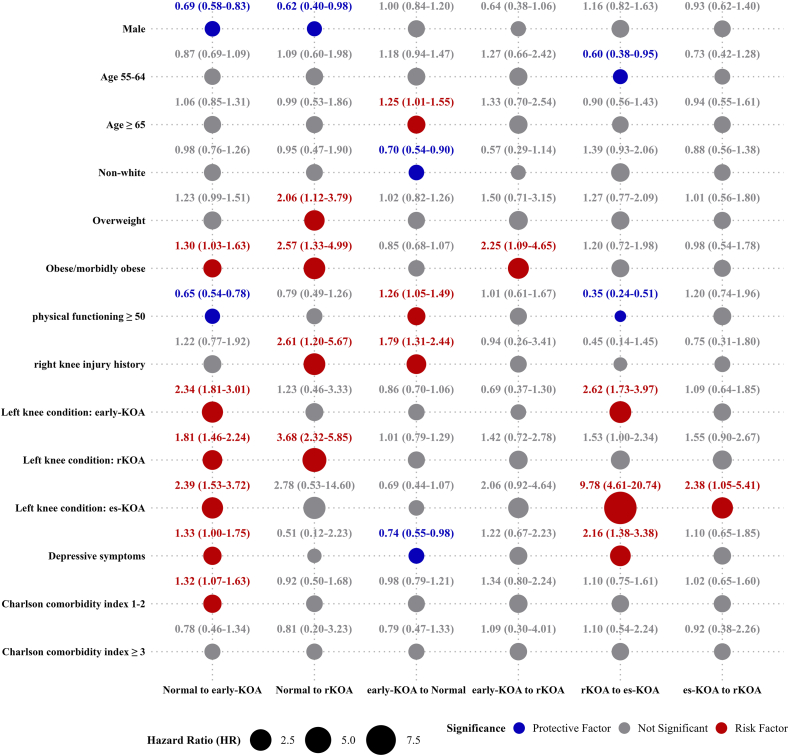
Effects of multifactorial covariates on transitions between KOA states. HRs and 95 ​% confidence intervals illustrate the effects of covariates on state transitions among KOA categories. Blue dots represent protective factors, red dots indicate risk factors, and gray dots indicate non-significant associations. The size of the dots reflects the magnitude of the HR.

Fig. 4 illustrates the effects of various multifactorial covariates on transitions between different KOA states. For BMI, being overweight increased the risk of transitioning from normal to rKOA compared with normal/underweight individuals, with a hazard ratio (HR) of 2.06 (95 ​% CI: 1.12–3.79). The risk was even higher for obese or morbidly obese individuals, with Hazard ratios (HRs) of 2.57 (95 ​% CI: 1.33–4.99) for transitioning from normal to rKOA, 1.30 (95 ​% CI: 1.03–1.63) for transitioning from normal to early-KOA, and 2.25 (95 ​% CI: 1.09–4.65) for progressing from early-KOA to rKOA.

Compared to individuals with a normal left knee status, those with more severe left knee conditions face a higher risk of right knee deterioration. Among individuals with left knee early-KOA or es-KOA, the HRs for progression in the right knee from normal to early-KOA and from rKOA to es-KOA were 2.34 (95 ​% CI: 1.81–3.01) and 2.62 (95 ​% CI: 1.73–3.97), and 2.39 (95 ​% CI: 1.53–3.72) and 9.78 (95 ​% CI: 4.61–20.74), respectively. For those with left knee rKOA, the HRs for progression in the right knee from normal to early-KOA and from normal to rKOA were 1.81 (95 ​% CI: 1.46–2.24) and 3.68 (95 ​% CI: 2.32–5.85), respectively.

High physical functioning (≥ 50) was associated with a lower risk of transitions. The HRs for transitioning from normal to early-KOA and from rKOA to es-KOA were 0.65 (95 ​% CI: 0.54–0.78) and 0.35 (95 ​% CI: 0.24–0.51), respectively. Individuals with depressive symptoms showed higher risks of transitioning from normal to early-KOA and from rKOA to es-KOA, with HRs of 1.33 (95 ​% CI: 1.00–1.75) and 2.16 (95 ​% CI: 1.38–3.38), respectively. Additionally, individuals with depressive symptoms were less likely to recover from early-stage KOA to a normal state, with an HR of 0.74 (95 ​% CI: 0.55–0.98). Male individuals were less likely to transition from the normal state to early-KOA or rKOA compared with females, with HRs of 0.69 (95 ​% CI: 0.58–0.83) and 0.62 (95 ​% CI: 0.40–0.98), respectively. The HRs of covariates for transitions between states in the univariate MSM model are summarized in Table S5. The transition covariate effects using the K-L grades and covariates without carry-forward imputation are detailed in Figs. S4 and S5.

### 3.5 The transition probability in subgroup

**Fig. 5:**
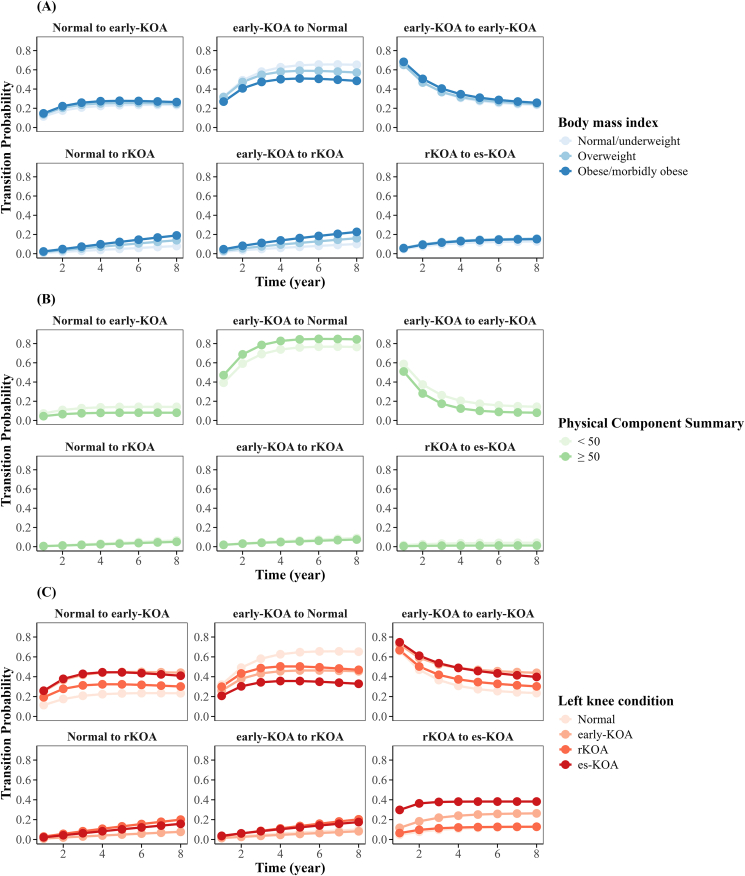
The estimated possibility of possible KOA state transitions within different subgroups. Estimated transition probabilities between different KOA states are presented, stratified by subgroups defined according to: (A) BMI, (B) physical component summary score, and (C) the condition of the left knee. The depicted transitions encompass: Normal to early-KOA, early-KOA to Normal, early-KOA to early-KOA, early-KOA to rKOA, early-KOA to es-KOA, and rKOA to es-KOA.

We evaluated the probabilities of transitions between KOA states across BMI, physical functioning, and left knee condition subgroups (Fig. 5). The obese/morbidly obese group had a higher probability of progression from normal to early-KOA and rKOA, early-KOA to rKOA, and rKOA to es-KOA, while the normal/underweight group showed a higher probability of recovery from early-KOA to normal. High physical functioning had lower probability of transitioning from normal to early-KOA and rKOA to es-KOA, and a higher likelihood of recovery from early-KOA to normal compared to those with scores < 50. Individuals with more severe left knee status had higher probability of progression across all stages in the right knee, while those with normal left knee status had a higher probability of recovery from early-KOA to normal in the right knee.

## 4 Discussion

This study analyzed the bidirectional transitions between distinct KOA states, with a focus on early-KOA and es-KOA, using an MSM model to evaluate transition intensity and probability. Our findings indicate that individuals in the early-KOA state are more likely to progress to rKOA, while also exhibiting potential for recovery. The protracted sojourn time in rKOA exemplifies the gradual progression of KOA. The transition between KOA states is influenced by specific factors, including the condition of the contralateral knee joint, BMI, sex, physical function, depressive symptoms, and knee injury history. Our conclusions were robust to alternative modelling choices regarding the es-KOA state, handling of regressive K–L grades, and covariate missingness. Differences across specifications were small and did not alter the direction of effects.

Our findings revealed that the transition intensity from early-KOA to rKOA (0.05, 95 ​% CI: 0.04–0.06) was higher than that from normal to rKOA (0.02, 95 ​% CI: 0.01–0.02), indicating that individuals in the early-KOA state face a higher risk of progressing to rKOA than those in the normal state and are also more prone to rapidly advancing to es-KOA. Furthermore, when estimating transition probabilities over a 1–8 year period, the probability of the early-KOA state reverting to the normal state is greater than that of progression, suggesting an opportunity for early intervention. Therefore, early-KOA is a concept that requires further refinement in order to identify a homogeneous subset of individuals exhibiting symptoms or functional limitations prior to the onset of significant radiographic structural changes (K-L grade < 2), and to distinguish them from those with established rKOA [9,23,24].

The prolonged sojourn time in rKOA (15.17 years) suggests its role as a pivotal, persistent state in KOA progression, consistent with longitudinal studies reporting slow radiographic progression in established osteoarthritis [25]. In contrast, the shorter sojourn time (2.76 years) and higher probability of recovery in individuals with es-KOA may reflect the tendency for many individuals to reach the point of indication for joint replacement surgery without actually undergoing the procedure, as pain subsides either spontaneously or with the use of analgesics, thus returning to a milder state [26]. Since not all KOA patients progress to the stage requiring joint replacement, this suggests that improvements in knee symptoms in es-KOA patients may delay the need for knee arthroplasty [17], avoiding the “final death” of the joint that necessitates surgery.

Individuals with more severe contralateral knee status in our study demonstrated elevated risks of progression to advanced states in the target knee. The presence of contralateral rKOA was associated with a HR of 3.68 (95 ​% CI: 2.32–5.85) for transitioning from normal to rKOA in the target knee, while contralateral early-KOA or es-KOA further increased the probability of progression to es-KOA. The strong association between contralateral knee status and ipsilateral progression underscores KOA as a systemic disorder rather than an isolated joint phenomenon. Shared biomechanical stressors, genetic predispositions, or systemic inflammation may drive this bilateral progression[[27], [28], [29]]. Unilateral strength training in patients with KOA has been shown to induce cross-education effects, enhancing strength and neuromuscular function in the contralateral limb, which can improve function and potentially slow the progression of KOA [30].

The relationship between BMI and progression risks reinforces obesity's dual role in KOA pathogenesis: mechanical overload and adipokine-driven inflammation [31]. A recent study suggests that leptin, as an adipokine, influences the pathogenesis of OA, implicating that OA is a systemic disease of adipose tissue [32]. Targeted weight loss interventions could attenuate both pathways, potentially decelerating transitions to advanced states. Male sex serves as a protective factor against the progression from normal to early KOA and rKOA. Compared to men, women tend to experience more severe pain and inflammation, greater reduction in cartilage volume, increased physical difficulties, and smaller joint parameters and dimensions [33,34], female may be structurally more vulnerable and at greater risk of OA [35]. Therefore, gender stratification is essential in both observational studies and interventions, with a particular emphasis on women. High physical function emerged as protective, likely due to stronger muscle strength and enhanced joint stability, which contribute to the prevention of KOA progression [36,37]. Conversely, functional decline may accelerate cartilage degradation through aberrant joint loading. These observations advocate for early incorporation of resistance training in at-risk populations.

Additionally, our analysis revealed that depressive symptoms were associated with impaired recovery from early-KOA (HR: 0.74, 95 ​% CI: 0.55–0.98) and an increased risk of progression to advanced stages. This may stem from the exacerbated pain severity frequently observed in individuals with depressive symptoms, highlighting the critical need to integrate mental health support into comprehensive KOA management frameworks [38]. A history of knee joint injury is a significant risk factor for the progression to rKOA. However, individuals with a history of knee joint injury are more likely to recover from early KOA to a normal state, possibly due to their enhanced understanding of pain management. Knee injuries severe enough to impair joint structure may compromise structural integrity, alter joint biomechanics, compress joint tissues, and increase the risk of KOA [[39], [40], [41]], with approximately half of affected individuals developing rKOA within 10–15 years post-injury [42].

Collectively, these findings delineate inter-related biomechanical, inflammatory, and psychosocial pathways that accelerate or mitigate KOA progression. Individuals with worse contralateral knee condition, high BMI, low physical function, or depressive symptoms represent high-risk groups who may benefit from intensified monitoring and early multimodal intervention. Incorporating weight reduction, resistance training, and psychosocial support into preventive strategies may delay progression to severe KOA and promote recovery during the potentially reversible stage. Incorporating these risk dimensions into trial design could enrich recruitment for disease-modifying interventions, Embedding such dynamic risk models into longitudinal monitoring frameworks may ultimately advance precision prevention and personalized management of KOA.

While this study provides novel insights into the dynamics of KOA progression, several limitations should be noted. First, given the predominance of White, US-based participants in OAI, the transportability of these transition patterns and covariate effects to underrepresented populations remains uncertain and warrants validation in multi-ethnic, non-US cohorts. Second, the conservative imputation of regressive K-L grades, while mitigating misclassification, may underestimate transient improvements in radiographic severity. Third, the inclusion and exclusion criteria of individuals exhibited notable demographic and clinical differences, which could introduce bias in the findings. Additionally, the exclusion of individuals who underwent knee arthroplasty during follow-up may have led to an overestimation of the transition probability for es-KOA recovery.

## Contributions

All authors were involved in the drafting or critical revision of the manuscript for important intellectual content, and provided final approval for publication of the article. It is worth noting that Pro. Lu had full access to all of the data in the study, and assumes responsibility for the integrity and accuracy of the data analysis.

Conceptualization. Aerman Nuer, Yasi Yang, Boran Sun, Wenli Lu.

Formal analysis. Aerman Nuer, Yasi Yang, Boran Sun, Yunhan Wang.

Methodology. Aerman Nuer, Wenbo Xiao.

Writing - original draft. Aerman Nuer, Yasi, Yang.

Writing – review and editing. Wenli Lu, Shu Wang, Lei Wang.

Final approval of the article. Wenli Lu, Shu, Wang, Lei Wang.

## Ethical statement

This study utilized publicly available, de-identified OAI data. As a secondary analysis, it was deemed exempt from separate institutional review board approval. The original OAI study received ethical approval and informed consent, with robust oversight provided by an independent Observational Study Monitoring Board, appointed by the National Institute of Arthritis and Musculoskeletal and Skin Diseases (NIAMS) at the National Institutes of Health from 2002 to 2014, ensuring participant safety and ethical conduct. This study was conducted in accordance with the ethical principles of the Declaration of Helsinki.

## Funding

This research did not receive any specific grant from funding agencies in the public, commercial, or not-for-profit sectors.

## Availability of data and materials

The data utilized in this study are publicly available for download at the following website: https://nda.nih.gov/oai.

## Conflicts of interest

All authors declare that they have no known competing financial interests or personal relationships that could have appeared to influence the work reported in this paper.
