# Supplementary material for: Progression and influencing factors of knee osteoarthritis based on a multi-state Markov model: Data from OAI
Source: Osteoarthr Cartil Open. 2025 Nov 13;8(1):100703. doi: 10.1016/j.ocarto.2025.100703 (PMC12681718; doi:10.1016/j.ocarto.2025.100703)
Supplement: Multimedia component 1 [file mmc1.docx]

**Supplementary material**

**Figure S1:** The Flow chart of participant selection

**Table S1**: The comparison of characteristics between enrolled individuals and excluded individuals

**Table S2**: The distribution of different states in each follow-up

**Table S3**: Observed numbers of states transitions from one follow-up to next follow-up

**Figure S2**: The compliance of each state observation rate with prediction rate

**Table S4**: Transition intensity between different states

**Figure S3**: Model-estimated transition probabilities over follow-up interval

**Table S5**: Model-estimated transition probabilities over follow-up interval

**Table S6**: Effects of the covariate on transitions among KOA states transitions

**Table S7**: Transition intensity between different states with es-KOA as an absorbing state

**Table S8**: Model-estimated transition probabilities over follow-up interval with es-KOA as an absorbing state

**Table S9**: Transition intensity between different states with raw K-L grades

**Table S10**: Model-estimated transition probabilities over follow-up interval with raw K-L grades

**Figure S4**: Effects of multifactorial covariates on transitions between KOA states with raw K-L grades

**Figure S5**: Effects of multifactorial covariates on transitions between KOA states without the use of covariate carry-forward imputation

**
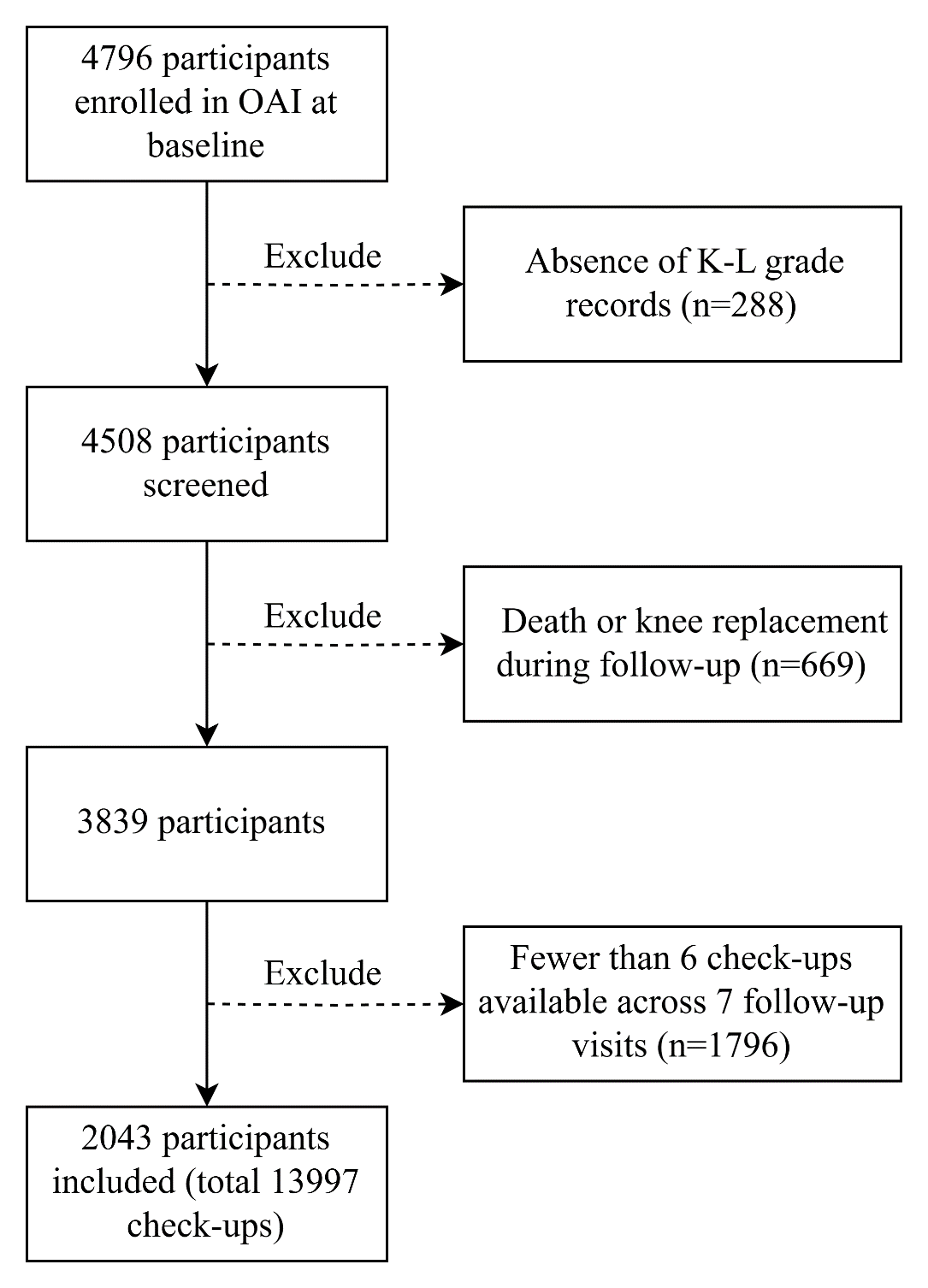
**

**Figure S1** The Flow chart of participant selection

**Table S1** The comparison of characteristics between enrolled individuals and excluded individuals

| **Characteristics** | **Exclude**  **(n = 2753)** | **Enrolled**  **(n = 2043)** | ***P*-value** |
| --- | --- | --- | --- |
| sex |  |  | <0.001 |
| Female | 1681 (61.1) | 1123 (55.0) |  |
| Male | 1072 (38.9) | 920 (45.0) |  |
| Age |  |  | <0.001 |
| < 55 | 697 (25.3) | 719 (35.2) |  |
| 55-64 | 872 (31.7) | 686 (33.6) |  |
| ≥ 65 | 1184 (43.0) | 638 (31.2) |  |
| Race |  |  | <0.001 |
| White | 2036 (74.0) | 1754 (85.9) |  |
| *Others | 717 (26.0) | 289 (14.1) |  |
| Marital status |  |  | <0.001 |
| married | 1748 (63.5) | 1430 (70.0) |  |
| Never married/divorced/separated/widowed | 971 (35.3) | 606 (29.7) |  |
| Body mass index |  |  | <0.001 |
| Normal/underweight | 558 (20.3) | 590 (28.9) |  |
| Overweight | 1050 (38.1) | 827 (40.5) |  |
| Obese/morbidly obese | 1142 (41.5) | 625 (30.6) |  |
| Education |  |  | <0.001 |
| Low education | 549 (19.9) | 226 (11.1) |  |
| Medium education | 1243 (45.2) | 904 (44.2) |  |
| High education | 927 (33.7) | 906 (44.3) |  |
| Pay for work |  |  | <0.001 |
| No | 1193 (43.3) | 657 (32.2) |  |
| Yes | 1558 (56.6) | 1385 (67.8) |  |
| Physical Component Summary |  |  | <0.001 |
| < 50 | 1453 (52.8) | 685 (33.5) |  |
| ≥ 50 | 1248 (45.3) | 1348 (66.0) |  |
| Smoking |  |  | 0.003 |
| Never | 2209 (80.2) | 1582 (77.4) |  |
| Current/former/current but not regular | 502 (18.2) | 446 (21.8) |  |
| Drinking |  |  | <0.001 |
| Never | 605 (22.0) | 328 (16.1) |  |
| < 7/week | 1740 (63.2) | 1440 (70.5) |  |
| ≥ 7/week | 373 (13.5) | 268 (13.1) |  |
| Left Knee condition |  |  | <0.001 |
| Normal | 646 (23.5) | 1197 (58.6) |  |
| early-KOA | 293 (10.6) | 436 (21.3) |  |
| rKOA | 1130 (41.0) | 346 (16.9) |  |
| es-KOA | 371 (13.5) | 64 (3.1) |  |
| Family knee replacement history |  |  | 0.627 |
| No | 2343 (85.1) | 1727 (84.5) |  |
| Yes | 375 (13.6) | 288 (14.1) |  |
| Right knee injury history |  |  | 0.084 |
| No | 1948 (70.8) | 1492 (73.0) |  |
| Yes | 805 (29.2) | 551 (27.0) |  |
| Diabetes |  |  | <0.001 |
| No | 2418 (87.8) | 1893 (92.7) |  |
| Yes | 249 (9.0) | 113 (5.5) |  |
| Hypertension |  |  | <0.001 |
| No | 1259 (45.7) | 1084 (53.1) |  |
| Yes | 1493 (54.2) | 959 (46.9) |  |
| Depressive symptoms |  |  | <0.001 |
| No | 2390 (86.8) | 1861 (91.1) |  |
| Yes | 316 (11.5) | 164 (8.0) |  |
| Charlson comorbidity index |  |  | <0.001 |
| 0 | 1952 (70.9) | 1613 (79.0) |  |
| 1-2 | 650 (23.6) | 370 (18.1) |  |
| ≥3 | 103 (3.7) | 42 (2.1) |  |

*The “other” race category encompasses participants identifying as American Indian/Alaska Native; Asian; Hawaiian or Pacific Islander; Black or African American; More than one race; as well as those whose race was Unknown or not reported, or reported as Other/Other Non-White. This aggregation was performed due to small sample sizes within these individual groups.

**Table S2** The distribution of different states in each follow-up

| **Time (year)** | **Normal, n (%)** | **Early-KOA, n (%)** | **rKOA, n (%)** | **Es-KOA, n (%)** | **Total records** |
| --- | --- | --- | --- | --- | --- |
| **baseline** | 1151(56.3) | 439(21.5) | 401(19.6) | 52(2.5) | 2043 |
| **1-year** | 1160(57.8) | 346(17.2) | 451(22.5) | 50(2.5) | 2007 |
| **2-year** | 1099(55.2) | 360(18.1) | 466(23.4) | 66(3.3) | 1991 |
| **3-year** | 1046(52.2) | 386(19.3) | 510(25.5) | 60(3.0) | 2002 |
| **4-year** | 1037(51.6) | 374(18.6) | 524(26.1) | 75(3.7) | 2010 |
| **6-year** | 961(49.1) | 337(17.2) | 567(29.0) | 92(4.7) | 1957 |
| **8-year** | 970(48.8) | 323(16.3) | 592(29.8) | 102(5.1) | 1987 |

**Table S3** Observed numbers of states transitions from one follow-up to next follow-up

|  | | **Post-transition, n (%)** | | | |
| --- | --- | --- | --- | --- | --- |
|  |  | **Normal** | **Early-KOA** | **rKOA** | **Es-KOA** |
| **Pre-transition,**  **n (%)** | **Normal** | 5512 (85.7) | 765 (11.9) | 147 (2.3) | 10 (0.2) |
|  | **Early-KOA** | 761 (34.0) | 1361 (60.9) | 88 (3.9) | 26 (1.2) |
|  | **KOA** | 0 (0.0) | 0 (0.0) | 2729 (94.2) | 169 (5.8) |
|  | **Es-KOA** | 0 (0.0) | 0 (0.0) | 146 (37.8) | 240 (62.2) |


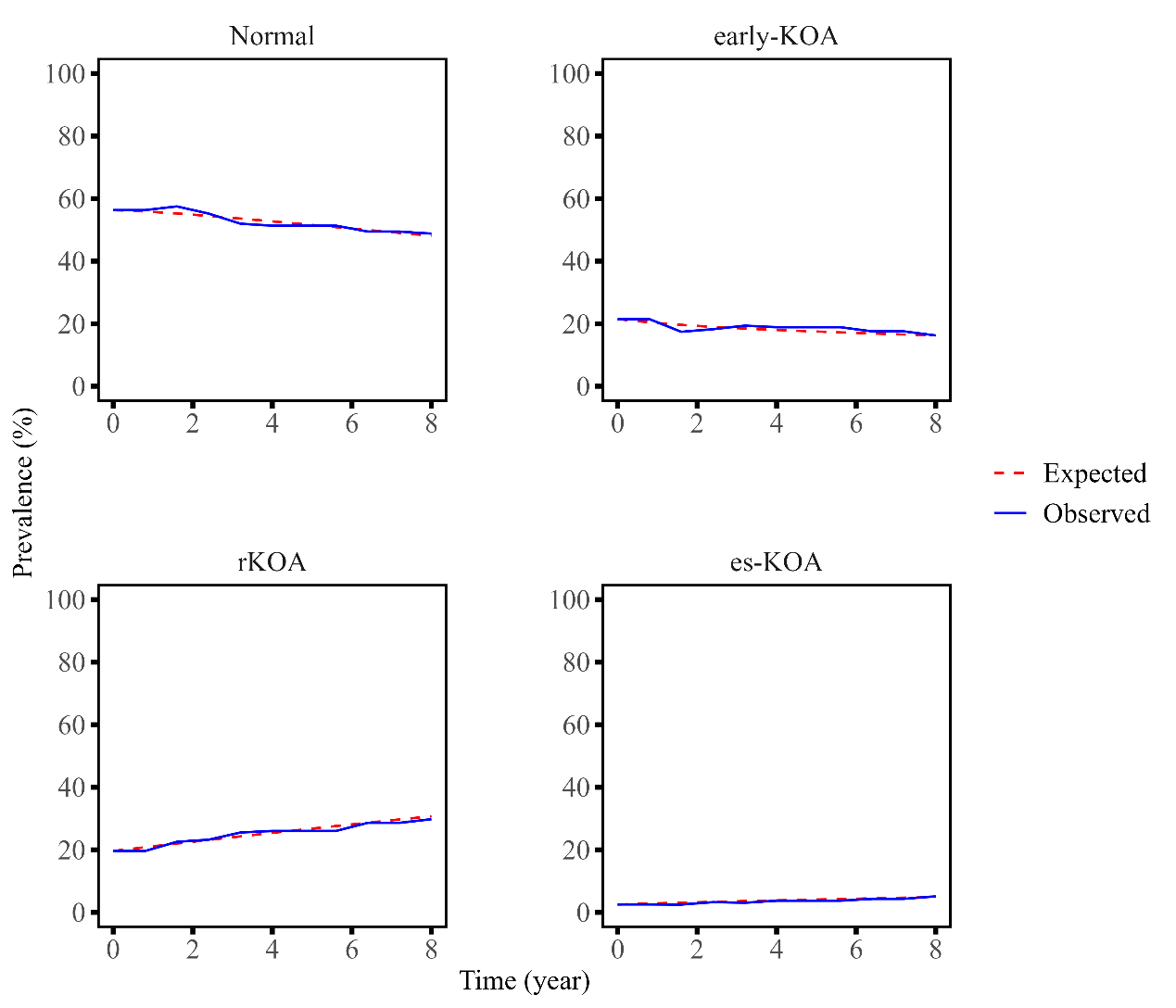


**Figure S2** The compliance of each state observation rate with prediction rate.

**Table S4** Transition intensity between different states

| **From/To** | Normal | early-KOA | rKOA | es-KOA |
| --- | --- | --- | --- | --- |
| Normal | -0.15(-0.16, -0.14) | 0.14(0.13, 0.15) | 0.02(0.01, 0.02) | 0 |
| early-KOA | 0.38(0.36, 0.42) | -0.43(-0.46, -0.40) | 0.05(0.04, 0.06) | 0 |
| rKOA | 0 | 0 | -0.07(-0.08, -0.06) | 0.07(0.06, 0.08) |
| es-KOA | 0 | 0 | 0.36(0.31, 0.43) | -0.36(-0.43, -0.31) |
| Sojourn Time (years) | 6.54(6.09, 7.02) 6.54(6.09, 7.02) | 2.33(2.17, 2.51) | 15.17(13.10, 17.57) | 2.76(2.33, 3.27) |


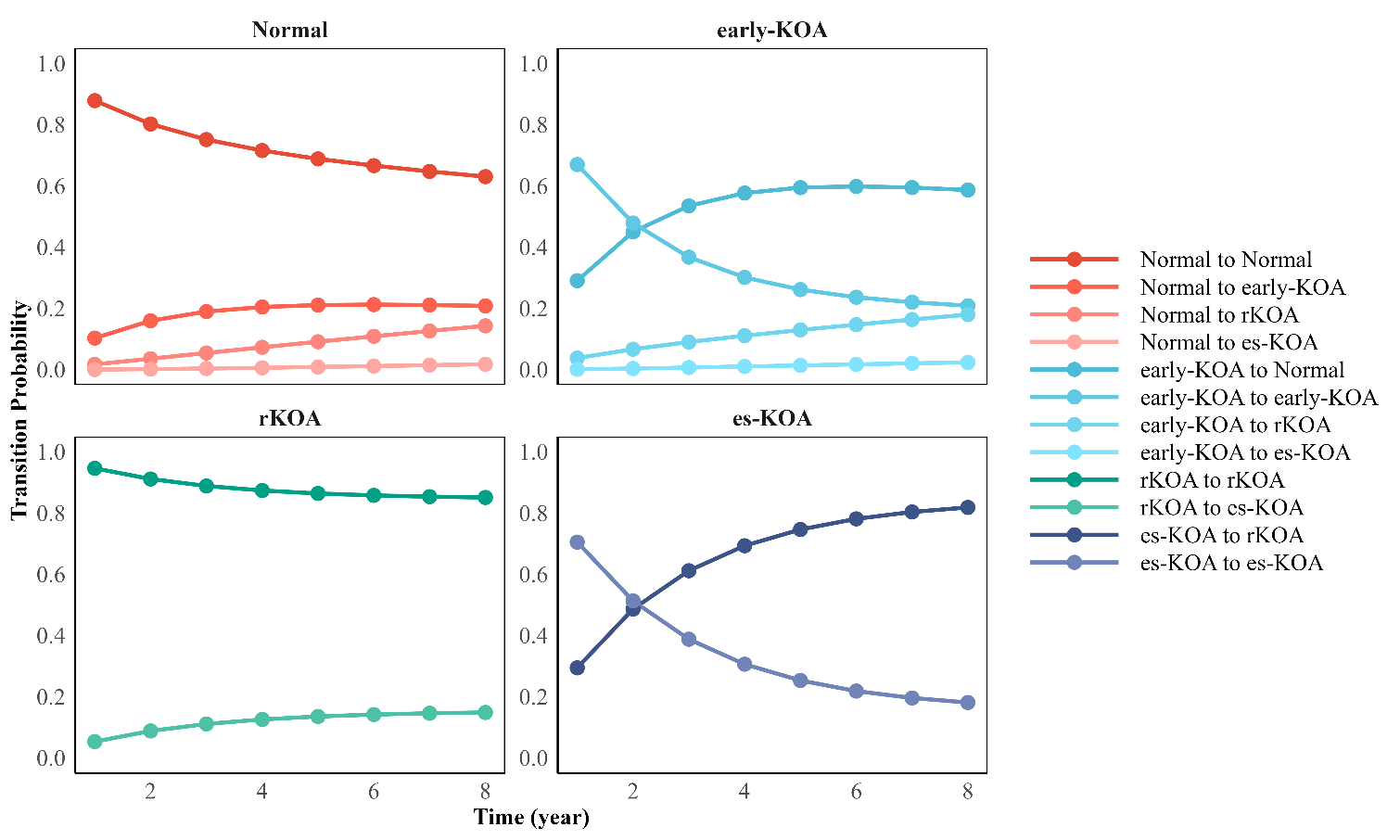


**Figure S3** Model-estimated transition probabilities over follow-up interval.

**Table S5** Model-estimated transition probabilities over follow-up interval.

| **Time**  **(year)** | **Normal**  **→**  **Normal** | **Normal**  **→**  **Early-KOA** | **Normal**  **→**  **rKOA** | **Normal**  **→**  **Es-KOA** | **Early-KOA →**  **Normal** | **Early-KOA →**  **Early-KOA** | **Early-KOA**  **→**  **rKOA** | **Early-KOA →**  **Es-KOA** | **rKOA**  **→**  **rKOA** | **Es-KOA**  **→**  **rKOA** | **Es-KOA**  **→**  **Es-KOA** |
| --- | --- | --- | --- | --- | --- | --- | --- | --- | --- | --- | --- |
| 1 | 0.879 | 0.103 | 0.017 | 0.001 | 0.291 | 0.67 | 0.038 | 0.001 | 0.946 | 0.295 | 0.705 |
| 2 | 0.802 | 0.160 | 0.036 | 0.002 | 0.450 | 0.479 | 0.067 | 0.004 | 0.911 | 0.487 | 0.513 |
| 3 | 0.752 | 0.190 | 0.055 | 0.004 | 0.535 | 0.367 | 0.090 | 0.007 | 0.889 | 0.612 | 0.388 |
| 4 | 0.716 | 0.205 | 0.073 | 0.006 | 0.577 | 0.301 | 0.111 | 0.011 | 0.874 | 0.693 | 0.307 |
| 5 | 0.689 | 0.211 | 0.091 | 0.009 | 0.595 | 0.262 | 0.130 | 0.014 | 0.864 | 0.747 | 0.253 |
| 6 | 0.667 | 0.213 | 0.109 | 0.012 | 0.599 | 0.237 | 0.147 | 0.017 | 0.858 | 0.781 | 0.219 |
| 7 | 0.648 | 0.211 | 0.126 | 0.015 | 0.595 | 0.220 | 0.164 | 0.021 | 0.854 | 0.804 | 0.196 |
| 8 | 0.631 | 0.208 | 0.143 | 0.018 | 0.587 | 0.209 | 0.180 | 0.024 | 0.851 | 0.819 | 0.181 |

**Table S6** Effects of the covariate on transitions among KOA states transitions

| **Covariates** | **Hazard Ratio (95% CI)** | | | | | |
| --- | --- | --- | --- | --- | --- | --- |
|  | **Normal→**  **Early-KOA** | **Normal→**  **rKOA** | **Early-KOA→**  **Normal** | **Early-KOA→**  **rKOA** | **rKOA→**  **Es-KOA** | **Es-KOA→**  **rKOA** |
| **Sex** |  |  |  |  |  |  |
| Female | Reference |  |  | Reference |  |  |
| Male | **0.73 (0.63-0.86)** | 0.69 (0.46-1.03) | 1.02 (0.87-1.19) | 0.77 (0.49-1.19) | 0.85 (0.63-1.14) | 0.87 (0.62-1.23) |
| **Age** |  |  |  |  |  |  |
| <55 | Reference |  |  | Reference |  |  |
| 55-64 | 1.12 (0.92-1.37) | 1.23 (0.70-2.15) | 1.21 (0.99-1.47) | 1.23 (0.73-2.07) | 0.80 (0.53-1.20) | 0.93 (0.60-1.44) |
| ≥65 | 0.98 (0.80-1.20) | 1.36 (0.78-2.36) | 1.18 (0.97-1.44) | 1.12 (0.65-1.95) | **0.58 (0.39-0.86)** | 0.80 (0.51-1.24) |
| **Race** |  |  |  |  |  |  |
| White | Reference |  |  | Reference |  |  |
| *Others | 1.19 (0.95-1.49) | 1.17 (0.64-2.15) | **0.63 (0.51-0.79)** | 0.81 (0.47-1.40) | **1.84 (1.31-2.57)** | 0.86 (0.60-1.23) |
| **Marital status** |  |  |  |  |  |  |
| married | Reference |  |  | Reference |  |  |
| Never married/Divorced/Separated/widowed | 1.03 (0.81-1.30) | 1.70 (1.16-2.50) | 0.93 (0.74-1.16) | 0.59 (0.34-1.04) | 0.92 (0.62-1.35) | 0.64 (0.40-1.03) |
| **Body mass index** |  |  |  |  |  |  |
| Normal and underweight | Reference |  |  | Reference |  |  |
| Overweight | 1.17 (0.97-1.42) | **2.13 (1.22-3.71)** | 0.87 (0.72-1.06) | 1.53 (0.80-2.92) | 1.49 (0.94-2.36) | 0.98 (0.58-1.65) |
| Obese and morbidly obese | **1.52 (1.25-1.86)** | **3.23 (1.83-5.71)** | **0.72 (0.59-0.88)** | **2.10 (1.13-3.90)** | **2.01 (1.28-3.16)** | 1.13 (0.69-1.87) |
| **Education** |  |  |  |  |  |  |
| Low education | Reference |  |  | Reference |  |  |
| Medium education | **0.75 (0.58-0.97)** | 0.86 (0.41-1.84) | 1.08 (0.84-1.39) | 0.85 (0.47-1.54) | 0.82 (0.53-1.28) | 0.78 (0.49-1.25) |
| High education | **0.57 (0.44-0.74)** | 0.82 (0.39-1.73) | 0.99 (0.77-1.28) | 0.71 (0.38-1.31) | 0.72 (0.46-1.14) | 0.83 (0.51-1.35) |
| **Pay for work** |  |  |  |  |  |  |
| No | Reference |  |  | Reference |  |  |
| Yes | 1.01 (0.86-1.19) | 0.69 (0.47-1.04) | 1.07 (0.91-1.25) | 1.22 (0.78-1.90) | **1.40 (1.03-1.89)** | 1.15 (0.81-1.64) |
| **Physical Component Summary** |  |  |  |  |  |  |
| ＜50 | Reference |  |  | Reference |  |  |
| ≥ 50 | **0.56 (0.48-0.66)** | **0.64 (0.42-0.96)** | **1.47 (1.26-1.73)** | 0.86 (0.55-1.34) | **0.24 (0.17-0.34)** | 1.09 (0.71-1.69) |
| **Smoking** |  |  |  |  |  |  |
| Never | Reference |  |  | Reference |  |  |
| Current/former/current but not regular | 0.95 (0.70-1.30) | 0.64 (0.36-1.15) | 0.78 (0.56-1.09) | 1.62 (1.00-2.62) | 1.09 (0.68-1.77) | **0.52 (0.18-1.47)** |
| **Drinking** |  |  |  |  |  |  |
| Never | Reference |  |  | Reference |  |  |
| <7week | 0.98 (0.70-1.38) | 1.21 (0.69-2.11) | 1.38 (0.96-1.98) | 0.77 (0.45-1.31) | 0.41 (0.24-0.70) | 0.63 (0.30-1.30) |
| ≥1/week | 1.02 (0.64-1.62) | 0.74 (0.32-1.71) | 1.47 (0.91-2.37) | 0.86 (0.40-1.87) | **0.39 (0.17-0.88)** | 0.86 (0.31-2.38) |
| **Left knee condition** |  |  |  |  |  |  |
| Normal | Reference |  |  | Reference |  |  |
| Early-KOA | **2.78 (2.18-3.55)** | 1.14 (0.37-3.47) | **0.81 (0.66-0.98)** | 0.81 (0.45-1.46) | **3.90 (2.64-5.74)** | 1.23 (0.75-2.03) |
| rKOA | **1.88 (1.53-2.30)** | **4.23 (2.73-6.55)** | 0.99 (0.79-1.25) | 1.56 (0.82-2.95) | **1.79 (1.21-2.66)** | 1.59 (0.97-2.61) |
| Es-KOA | **3.22 (2.21-4.69)** | **4.39 (1.40-13.79)** | **0.61 (0.40-0.95)** | **2.26 (1.07-4.80)** | **14.65 (7.80-27.52)** | 1.90 (0.99-3.64) |
| **Family knee replacement history** |  |  |  |  |  |  |
| No | Reference |  |  | Reference |  |  |
| Yes | **1.24 (1.01-1.52)** | 0.81 (0.43-1.50) | 1.03 (0.84-1.26) | 1.19 (0.72-1.96) | 0.92 (0.63-1.36) | 0.69 (0.44-1.09) |
| **Right knee injury history** |  |  |  |  |  |  |
| No | Reference |  |  | Reference |  |  |
| Yes | 1.21 (0.79-1.83) | **2.31 (1.08-4.94)** | **1.77 (1.33-2.35)** | 1.04 (0.36-3.02) | 0.46 (0.17-1.24) | 0.85 (0.37-1.92) |
| **Depressive symptoms** |  |  |  |  |  |  |
| No | Reference |  |  | Reference |  |  |
| Yes | **1.66 (1.29-2.15)** | 0.48 (0.12-1.98) | **0.68 (0.53-0.88)** | 1.28 (0.77-2.14) | **2.72 (1.84-4.02)** | 0.96 (0.62-1.48) |
| **Diabetes** |  |  |  |  |  |  |
| No | Reference |  |  | Reference |  |  |
| Yes | 1.29 (0.92-1.80) | 1.40 (0.69-2.84) | **0.52 (0.35-0.79)** | 1.01 (0.51-1.98) | 1.24 (0.74-2.05) | 0.76 (0.42-1.35) |
| **Hypertension** |  |  |  |  |  |  |
| No | Reference |  |  | Reference |  |  |
| Yes | 1.06 (0.91-1.24) | 1.16 (0.78-1.74) | 0.96 (0.82-1.12) | 1.41 (0.93-2.15) | 0.99 (0.73-1.32) | 0.85 (0.61-1.20) |
| **Charlson comorbidity index** |  |  |  |  |  |  |
| 0 | Reference |  |  | Reference |  |  |
| 1-2 | **1.30 (1.04-1.62)** | 1.09 (0.66-1.81) | **0.77 (0.61-0.97)** | 1.37 (0.89-2.13) | **1.89 (1.31-2.72)** | 1.11 (0.71-1.74) |
| ≥3 | 1.25 (0.73-2.16) | 1.03 (0.30-3.53) | 0.78 (0.47-1.29) | 0.89 (0.29-2.68) | **2.02 (1.04-3.94)** | 0.86 (0.38-1.95) |

Boldface indicates statistical significance (*P*<0.05).

**Table S7** Transition intensity between different states with es-KOA as an absorbing state.

| **From/To** | Normal | early-KOA | rKOA | es-KOA |
| --- | --- | --- | --- | --- |
| Normal | -0.15(-0.16, -0.14) | 0.14(0.13, 0.15) | 0.02(0.01, 0.02) | 0 |
| early-KOA | 0.38(0.36, 0.41) | -0.43(-0.46, -0.4) | 0.04(0.04, 0.05) | 0 |
| rKOA | 0 | 0 | -0.04(-0.05, -0.03) | 0.04(0.03, 0.05) |
| Sojourn Time (years) | 6.54(6.09, 7.02) | 2.33(2.17, 2.51) | 26.02(22.14, 30.59) | - |

**Table S8** Model-estimated transition probabilities over follow-up interval with es-KOA as an absorbing state.

| **Time**  **(year)** | **Normal**  **→**  **Normal** | **Normal**  **→**  **Early-KOA** | **Normal**  **→**  **rKOA** | **Normal**  **→**  **Es-KOA** | **Early-KOA →**  **Normal** | **Early-KOA →**  **Early-KOA** | **Early-KOA**  **→**  **rKOA** | **Early-KOA →**  **Es-KOA** | **rKOA**  **→**  **rKOA** | **Es-KOA**  **→**  **rKOA** |
| --- | --- | --- | --- | --- | --- | --- | --- | --- | --- | --- |
| 1 | 0.879 | 0.103 | 0.018 | 0 | 0.291 | 0.670 | 0.038 | 0.001 | 0.962 | 0.038 |
| 2 | 0.802 | 0.160 | 0.036 | 0.001 | 0.45 | 0.479 | 0.068 | 0.003 | 0.926 | 0.074 |
| 3 | 0.752 | 0.190 | 0.055 | 0.003 | 0.535 | 0.367 | 0.092 | 0.006 | 0.891 | 0.109 |
| 4 | 0.716 | 0.205 | 0.074 | 0.006 | 0.577 | 0.301 | 0.112 | 0.010 | 0.858 | 0.142 |
| 5 | 0.689 | 0.211 | 0.091 | 0.009 | 0.595 | 0.262 | 0.129 | 0.014 | 0.825 | 0.175 |
| 6 | 0.667 | 0.213 | 0.108 | 0.013 | 0.599 | 0.237 | 0.145 | 0.020 | 0.794 | 0.206 |
| 7 | 0.648 | 0.211 | 0.124 | 0.017 | 0.595 | 0.220 | 0.159 | 0.026 | 0.764 | 0.236 |
| 8 | 0.631 | 0.208 | 0.139 | 0.022 | 0.587 | 0.209 | 0.172 | 0.032 | 0.735 | 0.265 |

**Table S9** Transition intensity between different states with raw K-L grades.

| **From/To** | Normal | early-KOA | rKOA | es-KOA |
| --- | --- | --- | --- | --- |
| Normal | -0.15(-0.16--0.14) | 0.14(0.13-0.15) | 0.02(0.01-0.02) | 0 |
| early-KOA | 0.38(0.36-0.41) | -0.43(-0.46--0.4) | 0.05(0.04-0.06) | 0 |
| rKOA | 0 | 0 | -0.07(-0.08--0.06) | 0.07(0.06-0.08) |
| es-KOA | 0 | 0 | 0.39(0.33-0.48) | -0.39(-0.48--0.33) |
| Sojourn Time (years) | 6.54(6.09, 7.02) | 2.33(2.17, 2.5) | 14.02(11.90, 16.52) | 2.54(2.10, 3.07) |

**Table S10** Model-estimated transition probabilities over follow-up interval with raw K-L grades.

| **Time**  **(year)** | **Normal**  **→**  **Normal** | **Normal**  **→**  **Early-KOA** | **Normal**  **→**  **rKOA** | **Normal**  **→**  **Es-KOA** | **Early-KOA →**  **Normal** | **Early-KOA →**  **Early-KOA** | **Early-KOA**  **→**  **rKOA** | **Early-KOA →**  **Es-KOA** | **rKOA**  **→**  **rKOA** | **Es-KOA**  **→**  **rKOA** | **Es-KOA**  **→**  **Es-KOA** |
| --- | --- | --- | --- | --- | --- | --- | --- | --- | --- | --- | --- |
| 1 | 0.879 | 0.103 | 0.017 | 0.001 | 0.290 | 0.670 | 0.038 | 0.001 | 0.943 | 0.057 | 0.315 |
| 2 | 0.802 | 0.160 | 0.036 | 0.002 | 0.450 | 0.479 | 0.067 | 0.004 | 0.907 | 0.093 | 0.512 |
| 3 | 0.752 | 0.190 | 0.054 | 0.004 | 0.534 | 0.367 | 0.091 | 0.007 | 0.885 | 0.115 | 0.636 |
| 4 | 0.716 | 0.205 | 0.073 | 0.006 | 0.576 | 0.301 | 0.111 | 0.011 | 0.870 | 0.130 | 0.715 |
| 5 | 0.689 | 0.211 | 0.091 | 0.009 | 0.594 | 0.261 | 0.130 | 0.015 | 0.862 | 0.138 | 0.764 |
| 6 | 0.667 | 0.213 | 0.109 | 0.012 | 0.598 | 0.236 | 0.147 | 0.018 | 0.856 | 0.144 | 0.794 |
| 7 | 0.648 | 0.211 | 0.126 | 0.015 | 0.594 | 0.220 | 0.164 | 0.021 | 0.852 | 0.148 | 0.814 |
| 8 | 0.631 | 0.209 | 0.143 | 0.018 | 0.586 | 0.209 | 0.180 | 0.025 | 0.850 | 0.150 | 0.826 |


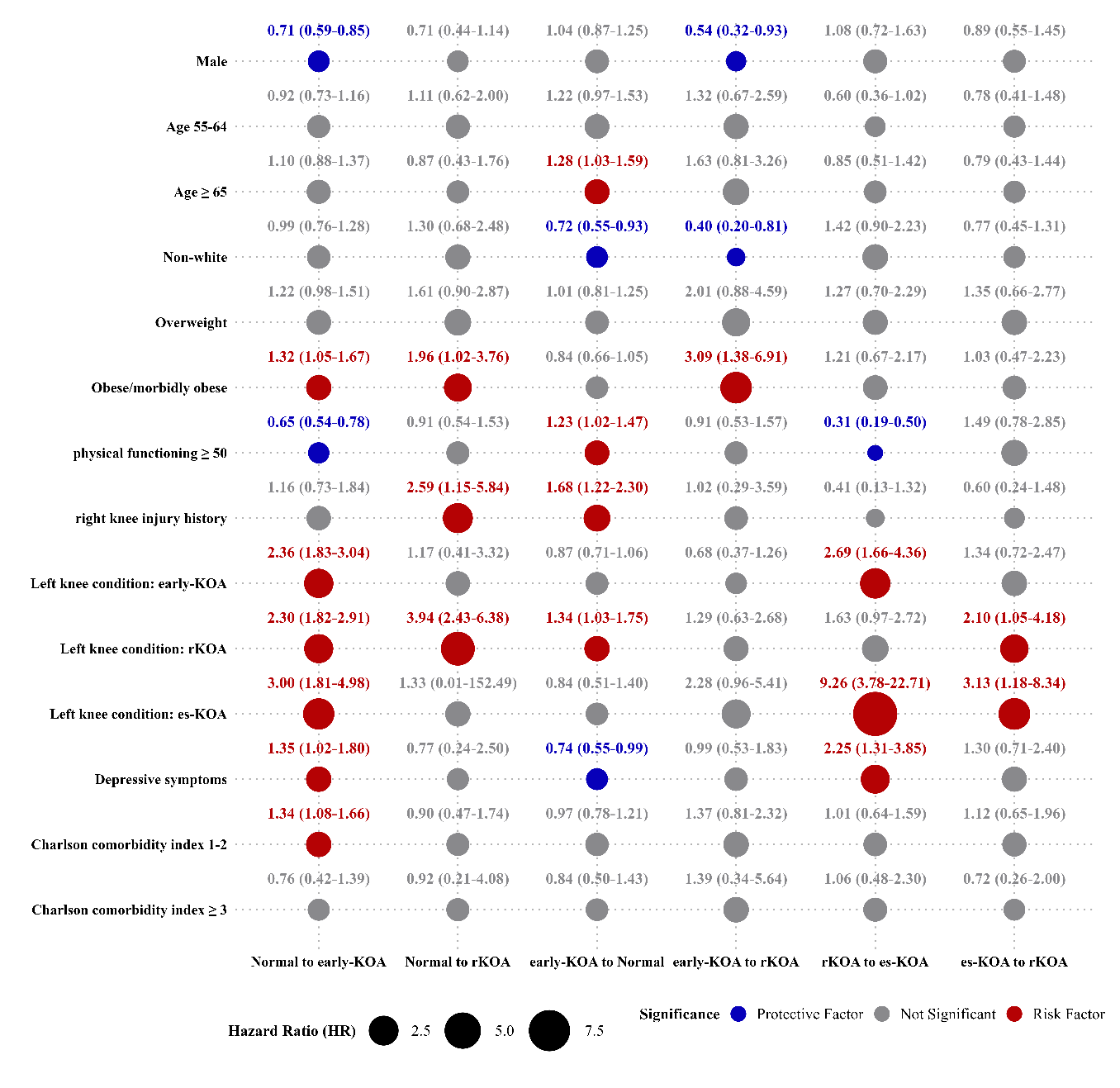


**Figure S4** Effects of multifactorial covariates on transitions between KOA states with raw K-L grades.


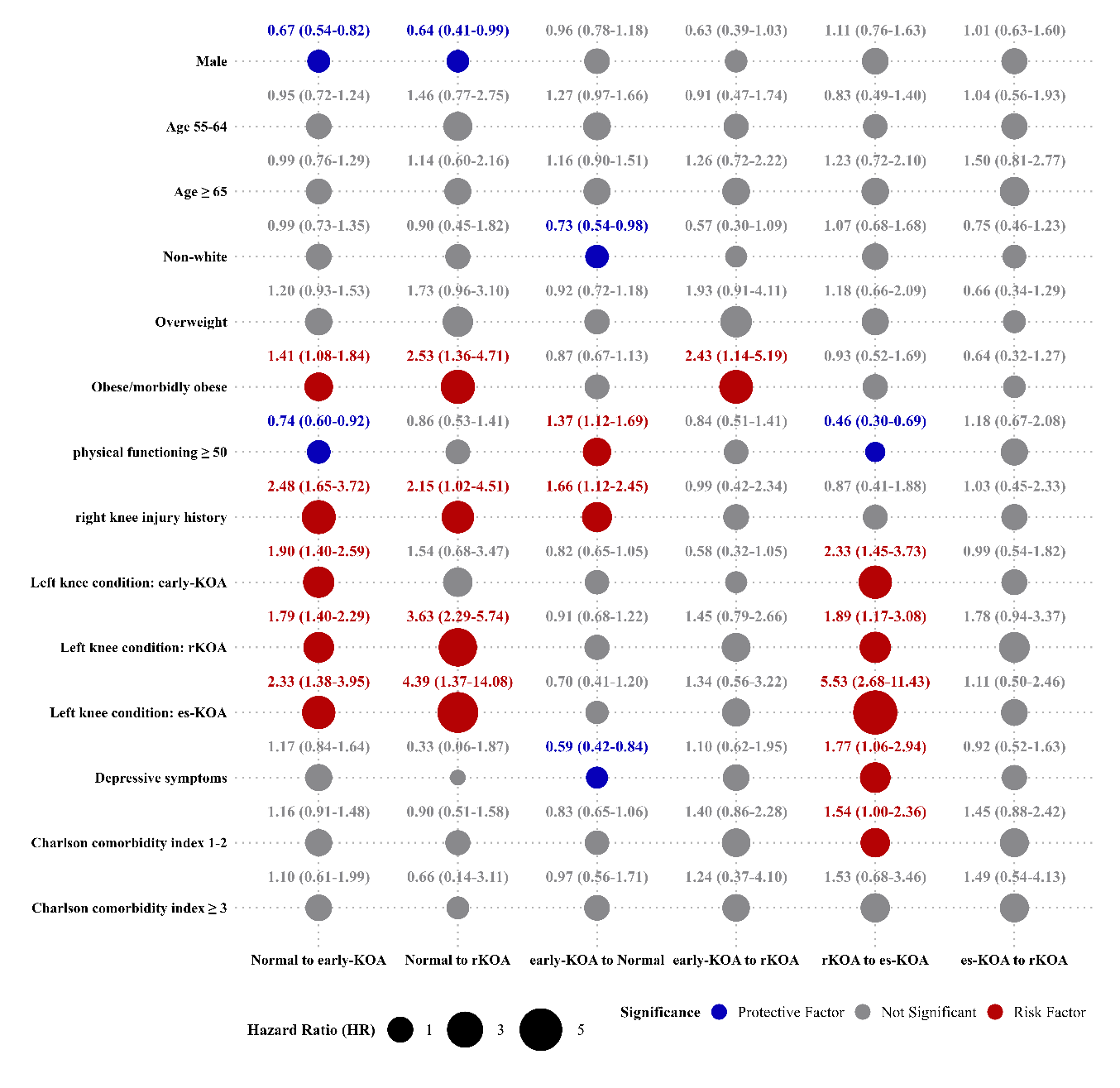


**Figure S5** Effects of multifactorial covariates on transitions between KOA states without the use of covariate carry-forward imputation.
